# Early-onset fetal growth restriction treated with the long-acting phosphodiesterase-5 inhibitor tadalafil: a case report

**DOI:** 10.1186/s13256-016-1098-x

**Published:** 2016-11-08

**Authors:** Mito Sakamoto, Kazuhiro Osato, Michiko Kubo, Masafumi Nii, Hiroaki Tanaka, Nao Murabayashi, Takashi Umekawa, Yuki Kamimoto, Tomoaki Ikeda

**Affiliations:** Mie University Hospital, 174-2 Edobashi, Tsu City, Mie Japan

**Keywords:** Tadalafil, Sildenafil, Fetal growth restriction, Oligohydramnios, Phosphodiesterase-5 inhibitor

## Abstract

**Background:**

Severe early-onset fetal growth restriction occurs in 0.4 % of all pregnancies, and the prognoses of these patients are dismal. Severely growth-restricted fetuses (far below 500 g) are thought to be nonviable. Since there have not been effective treatments for such fetal patients, obstetricians have simply tried to identify the optimal timing for their delivery. There are a few reports suggesting that the phosphodiesterase type 5 inhibitor sildenafil has some limited beneficial effects on fetal growth, but there are no such reports on tadalafil, another derivative phosphodiesterase type 5 inhibitor which has a much longer half-life than sildenafil. Here we present a case in which the administration of tadalafil to the mother revived the arrested growth and severe oligohydramnios of the very prematurely growth-restricted fetus.

**Case presentation:**

We describe a case of early-onset fetal growth restriction with oligohydramnios in a 41-year-old primigravida Japanese woman who was treated with tadalafil (20-mg tablet daily) from 22 weeks’ gestational age. Ten days after the initiation of the tadalafil therapy, the amniotic fluid level rose and the weight of the fetus began to increase. A 1024-g baby boy was delivered by cesarean at 32 weeks’ gestation. The z-score for fetal head circumference had increased from −2.2 to −1.2, whereas the z-score of the femur legth was decreased to −4.3, indicating that tadalafil preferentially increased the blood flow to important organs.

**Conclusions:**

We achieved two positive results by administering tadalafil to the mother carrying a severely growth-restricted fetus with oligohydramnios. First, the z-scores of head circumference and abdominal circumference had at first declined but started to rise after the tadalafil administration. Second, the amniotic fluid, which was emptied before the tadalafil treatment, recovered to normal range with this treatment. Tadalafil administration to mothers could be a promising therapy to reverse severe fetal growth restriction and oligohydramnios.

## Background

The management of fetal growth restriction is based on the prolongation of gestation long enough for fetal organs to mature while simultaneously preventing irreversible deterioration of the fetus’ well-being. This policy is used because there has been no treatment to reverse fetal growth *in utero* until now. The administration of aspirin or oxygen to the mother and in-patient bed rest for the mother were all found to have no beneficial effects on fetal growth in randomized control studies [1–3]. Several promising therapies are currently under development to reverse fetal growth restriction [4]. One of these therapies is sildenafil, a phosphodiesterase (PDE) 5 inhibitor that is used to treat both pulmonary hypertension and erectile dysfunction. In an open-label pilot study, treatment with the PDE5 inhibitor sildenafil seemed to be effective in increasing fetal abdominal circumference (AC) of patients with early-onset fetal growth restriction [5].

The PDE5 inhibitor tadalafil was developed as a medication that was longer acting than sildenafil (the biological half-life of tadalafil is 14 to 15 hours versus sildenafil’s 3 hours) for the treatment of pulmonary hypertension, erectile dysfunction, and prostatic hypertrophy. We used tadalafil successfully in the following single case of early-onset severe fetal growth restriction.

## Case presentation

At a 19-weeks gestational checkup of a post-*in vitro* fertilization pre-embryo transfer (IVF-ET) pregnancy of a 41-year-old primigravida Japanese woman, the result of a fetal chromosomal test was normal, and normal amniotic fluid was observed. At 22 weeks and 4 days, severe oligohydramnios was observed (the amniotic fluid index decreased to nearly 0) with the fetal bladder emptied. The estimated fetal weight was 309 g: −2.6 standard deviation (SD). As judged by the biparietal diameter (BPD), the growth of the fetus had been arrested by nearly 4 weeks.

**Fig. 1:**
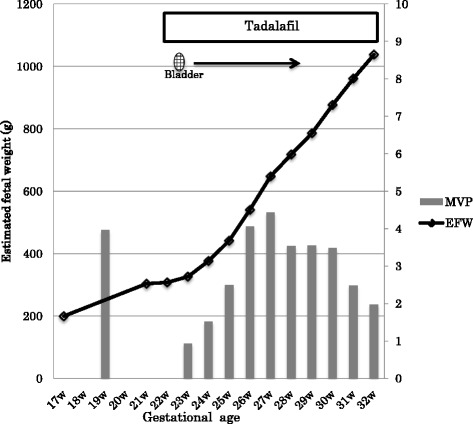
The estimated weight of the fetus increased, the fetal bladder dilated, and the amniotic fluid level increased after tadalafil therapy. *EFW* estimated fetal weight, *MVP* mean vertical pocket of amniotic fluid, *W* week

Our patient started to take a 20-mg tablet of tadalafil per day after getting permission from our hospital’s Institutional Review Board (Mie University Hospital approval #135-392), and informed consent for this treatment was obtained from our patient and her husband. Four days after the start of the daily tadalafil therapy, the fetal bladder started to dilate, and at 10 days of treatment the amniotic fluid level was increased (Fig. 1). The weight of the fetus started to rise, and the increase in the well-being of the fetus was confirmed by the biophysical score. The mother reported no side effects, including hypotension and headache. She did not develop pregnancy-induced hypertension at any time during the pregnancy.

The weight of the fetus increased by approximately 50 to 100 g per week. The amniotic fluid maximum vertical distance increased from 0.9 cm to 4.4 cm at 27 weeks’ gestational age, then decreased to <2 cm at 32 week’s gestation. At this time, fetal heart rate monitoring showed many variable decelerations, and the fetus was in the breech position. We informed our patient about this situation, and after careful consideration she and her husband chose to continue to the delivery. At 32 weeks 2 days, we performed a cesarean because the weight of the fetus had reached >1000 g, which is generally thought to guarantee intact survival [6]. At the moment of the cesarean delivery, the umbilical artery pulsatility index (PI) was 0.97, the middle cerebral artery PI was 1.48, and the ductus venosus wave was negative. A 1024-g baby boy was delivered without acidemia, with Apgar scores of 5 and 7 at 1 minute and 5 minutes after birth. The baby boy is now on an uneventful course at 3-months old.

**Fig. 2:**
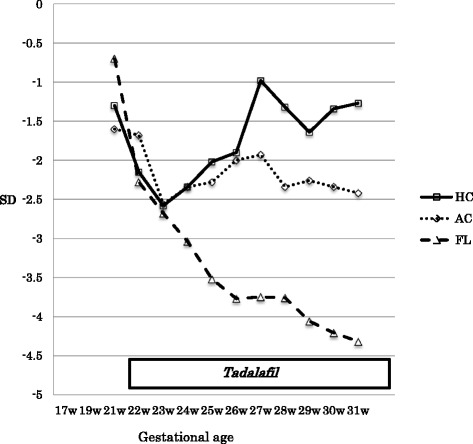
Change in the z-scores in head circumference, abdominal circumference, and femur length from before to after tadalafil therapy. *AC* abdominal circumference, *FL* femur length, *HC* head circumference, *SD* standard deviation, *W* week

Figure 2 shows the changes in z-scores of the size parameters of the fetus estimated by ultrasonography. Before the start of tadalafil therapy, the z-scores of head circumference (HC), AC, and femur length (FL) decreased to −2.5. After the completion of the 10-week tadalafil therapy, the HC z-score increased significantly to over −1.5 after 30 weeks’ gestation. The AC z-score kept up with the standard curve, remaining between −1.8 and −2.5. On the other hand, the FL z-score decreased consistently down to −4.3 at 31 gestational weeks.

**Fig. 3:**
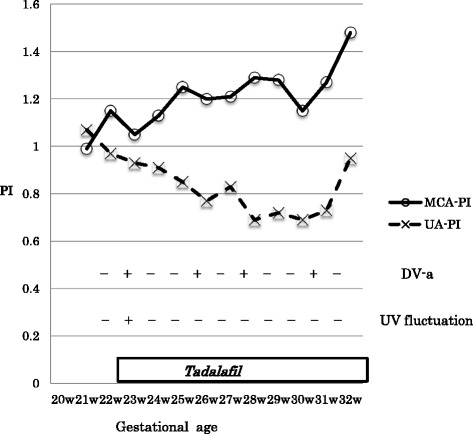
The parameters of the Doppler examination for fetal arteries and veins. *DV-a* (*rev*) reverse flow of ductus venosus a-wave, *MCA-PI* middle cerebral artery pulsatility index, *PI* pulsatility index, *UA-PI* umbilical artery pulsatility index, *UV* umbilical vein, *W* week

Figure 3 shows the changes in the Doppler parameters of the fetal arteries and veins. The umbilical artery PI decreased after the tadalafil treatment (from 1.11 to 0.69), although it was within the normal range throughout our patient’s pregnancy. The PI of the middle cerebral artery showed a spearing effect at first (1.15) and decreased temporarily (1.05) for 1 week after the initiation of the tadalafil treatment, then gradually increased to 1.5 at 32 weeks’ gestation. Fluctuations of the PI of the umbilical vein were observed before the tadalafil therapy, but were not seen 7 days after the completion of the therapy. The reverse flow of the ductus venosus seen before the therapy disappeared temporarily, but it appeared intermittently during the treatment course. As for the uterine artery velocimetry, the PI value was not significantly different before or after tadalafil treatment (0.79 and 0.62 respectively), and notching was not observed at any time.

A pathological examination of the placenta revealed excessive torsion of the cord, chorangiosis, and infarction, which suggested that placental hypoperfusion may have contributed to the growth of the fetus. There was no funisitis or chorioamnionitis.

## Discussion

This is the first report of tadalafil being used for the treatment of early-onset fetal growth restriction, to the best of our knowledge. Twenty mg per day of tadalafil — which is the same dosage as that used for the treatment of pulmonary hypertension — not only restarted the growth of the fetus but also improved the condition of the fetus, increasing fetal urine and amniotic fluid levels.

Tadalafil was developed in 2009 as a new PDE-5 inhibitor with a longer biological half-life than sildenafil, and it has recently become more widely used for the treatment of pulmonary hypertension, erectile dysfunction, and prostate hypertrophy. Tadalafil has an advantage in that it can be taken even on a full stomach, unlike sildenafil. To-date, case reports [7, 8] and a small pilot trial study [5] demonstrated that sildenafil had a beneficial effect on early-onset fetal growth restriction. von Dadelszen *et al*. [5] reported that their group (*n* = 10) of cases of fetal growth restriction treated with sildenafil showed significantly increased ACs compared with a group of retrospectively gathered cases of fetal growth restriction (*n* = 17). Theoretically, tadalafil seems to be a better choice than sildenafil because it can achieve a steadier blood concentration and effects.

The rationale for the use of tadalafil therapy in the present case – the reason why the fetus would have died without life-saving intervention – is twofold. First, early-onset fetal growth restriction that shows umbilical venous fluctuation and reverse ductus venosus flow, which were observed in this case, is the strongest predictive factor for intrauterine fetal death [9]. Second, almost one-half of the reported cases of early-onset fetal growth restriction were complicated with severe oligohydramnios and destined to die [10]. We believe that in the present case the tadalafil therapy contributed to the improved intrauterine environment and to the extension of the pregnancy to 32 week’s gestation, and finally to the uncomplicated post-delivery course.

It is interesting to note the different rates of the growth curves of the HC, AC, and FL z-scores in our patient (Fig. 2). The HC z-score increased significantly after the initiation of tadalafil therapy and finally became within normal range, whereas the FL z-score progressively worsened to −4.3. The AC z-score remained between −1.8 and −2.5. These findings indicate that tadalafil preferentially dilates the fetal arteries and distributes blood flow to more important organs such as the brain. In contrast, tadalafil might have increased the placental circulation through the internal iliac arteries at the expense of the blood flow of the external iliac arteries to inferior extremities.

The umbilical artery PI decreased after the initiation of tadalafil treatment in the present case, as Dastjerdi *et al*. reported for sildenafil [11], whereas the middle cerebral artery PI temporarily decreased for 1 week after the initiation of tadalafil treatment in our patient, unlike the Dastjerdi report. This result indicates that tadalafil was transferred through the placenta and dilated the cerebral arteries as well as placental arteries, even as its brain-sparing effect had already been exerted.

Another interesting finding in the present case was that the reverse flow of the ductus venosus appeared intermittently after the normalization that was induced by tadalafil therapy. This indicates that tadalafil dilated not only arteries but also veins of the placenta, which led to an increase in the venous flow to the heart, manifested as a reverse return of the ductus venosus.

A limitation of this case report is that it involves only one case, and thus no conclusions can be made regarding the best timing for tadalafil treatment or its potential maternal and fetal adverse effects (including long-term effects), or the evolution of Doppler parameters.

## Conclusions

Tadalafil administration to mothers could be a promising therapy to reverse severe fetal growth restriction and oligohydramnios. However, the present case highlights the need for further research about this use of tadalafil, with animal models and clinical studies.
